# Transporter gene variants influencing metformin pharmacokinetics and pharmacodynamics common in European populations: a pharmacogenetic narrative review

**DOI:** 10.3389/fphar.2026.1794405

**Published:** 2026-06-19

**Authors:** Natalie Mlcuchova, Bretislav Lipovy, Ondrej Zendulka, Jaroslav Janosek, Petra Borilova Linhartova

**Affiliations:** 1 RECETOX, Faculty of Science, Masaryk University, Brno, Czechia; 2 Department of Burns Medicine, Third Faculty of Medicine, Charles University and University Hospital Kralovske Vinohrady, Prague, Czechia; 3 Department of Pharmacology, Faculty of Medicine, Masaryk University, Brno, Czechia; 4 Centre for Health Research, Faculty of Medicine, University of Ostrava, Ostrava, Czechia

**Keywords:** Antidiabetics, personalized medicine, pharmacogenetics, pharmacology, single nucleotide variant

## Abstract

Metformin is a widely used drug with a relatively good efficacy in diabetes treatment, a good safety profile, and the potential for use in other indications. The variability in the individual responses to metformin therapy is partially determined by genetic factors. This narrative review aimed to summarize information on single nucleotide variant (SNVs) in genes for transporter proteins associated with metformin pharmacokinetics and pharmacodynamics and/or the occurrence of adverse effects. The Pharmacogenomics Knowledge Base (PharmGKB) and Web of Science databases were searched for metformin-associated gene variants that could affect its action. Seven transporter genes with twelve SNVs common in the European population were identified in the PharmGKB database, namely SNVs in genes *SLC22A1* (OCT1), *SLC22A2* (OCT2), *SLC22A3* (OCT3), *SLC22A4* (OCTN1), *SLC47A1* (MATE1), *SLC47A2* (MATE2-K)*,* and *SLC2A2* (GLUT2); it is worth noting that GLUT2 is not metformin transported but a glucose transporter and as such, it can also influence metformin action. Based on 63 retrieved studies, the association of individual SNVs with metformin effectiveness and adverse effects is discussed. In view of the high variability of study designs, populations, and reporting patterns, we also propose a framework for the design and reporting of metformin-associated pharmacogenetic studies, suggesting also that determining a complete set of these SNVs could help in comprehensive understanding of genetically conditioned individual responsiveness to metformin therapy, thus opening the path to maximizing the utilization of its positive effects while minimizing the risk of adverse effects. In addition, given the large variability in designs among studies, we also propose a framework for future studies on SNVs in metformin action-associated transporters that could improve comparability of future studies.

## 1 Introduction

Metformin is the drug of choice in patients with newly diagnosed Type 2 diabetes mellitus (T2DM) (Bailey, 2017; Flory and Lipska, 2019), typically administered in monotherapy along with recommendations for lifestyle modifications (American Diabetes Association, 2020). Besides regulating glucose metabolism, metformin was also shown to regulate lipid metabolism. Potential antitumor, antioxidant, cardio- and neuroprotective effects of metformin have been previously described (Patil et al., 2014; Argun et al., 2015; Tohamy et al., 2021; Sato et al., 2025), along with its use in the treatment of the polycystic ovary syndrome (PCOS) (Wang et al., 2017), dermatological disorders (Bubna, 2016), and in other applications. It has also been shown to be effective in the treatment of antipsychotic-induced weight gain in patients with schizophrenia and schizoaffective disorder (de Silva et al., 2016). The wide spectrum of metformin use is given by its low price, good safety profile, effective reduction of blood glucose, body weight neutrality (or loss), and cardioprotective effects (Sanchez-Rangel and Inzucchi, 2017).

Still, a relatively large group of patients do not respond to treatment (Rashid et al. reported in their study on 200 patients that 40.5% were non-responders [i.e., their glycated hemoglobin (HbA1c) levels had decreased by <0.8% from the baseline within 3 months of metformin therapy (Rashid et al., 2019)]. Others suffer from drug intolerance (20%), presenting with issues such as gastrointestinal complaints, taste disorders, and, in rare cases, vitamin B_12_ deficit or metformin-associated lactic acidosis (MALA) (Sanchez-Rangel and Inzucchi, 2017; Chowdhury et al., 2018; García-Calzón et al., 2020; Farooq et al., 2022; Nabrdalik et al., 2022). MALA is a rare but potentially fatal complication (mortality 3%–50%). Its risk is higher in patients with pre-existing conditions such as renal dysfunction or those taking additional medications that promote lactate accumulation. OCT2 (*SLC22A2*) variants, such as rs316019, have been reported to be possibly associated with altered metformin transport and might influence the risk of susceptibility to MALA (Hatmal et al., 2025). In 5% of patients, metformin use needs to be discontinued due to serious intolerance (Florez, 2017). The inter-individual as well as inter-populational differences in metformin pharmacokinetics or pharmacodynamics are, among other factors, given by genetic predispositions of the patient and/or the population. Understanding the multigenic innate characteristics of the given patient could help in personalizing metformin therapy.

Pharmacogenetic studies aim to determine the risk of non-response to therapy and the occurrence of adverse effects in predisposed individuals. Determining the genetic profile can help in patient classification into expected responders and non-responders; in other words, it can, based on the genetic data and clinical response to the drug, help identify patients who are likely to respond well to metformin treatment (Florez, 2017). Genotype-guided therapy could, therefore, help personalize the treatment from the perspectives of both metformin suitability and dosage at an individual-patient level, thus ensuring improved treatment results and reduced adverse side effects.

While most previous studies on pharmacogenetics of metformin focus on Asian or global cohorts, this narrative review brings a comprehensive overview of SNVs in transporter genes associated with metformin action that are selected with a particular emphasis on the variants common in European population. Further, considering the complexity of interactions among individual SNVs and the variability of designs and reporting patterns, we also aimed to propose a framework for performing future studies on SNVs associated with transporter genes influencing metformin action and suggest a set of promising candidate SNVs that should be always investigated in the European population. In effect, this would support better analysis of the interplay of these SNVs and, hopefully, become a basis for the personalized prediction of metformin effectiveness and adverse effects, thus improving the quality of metformin treatment.

## 2 Methods

**FIGURE 1:**
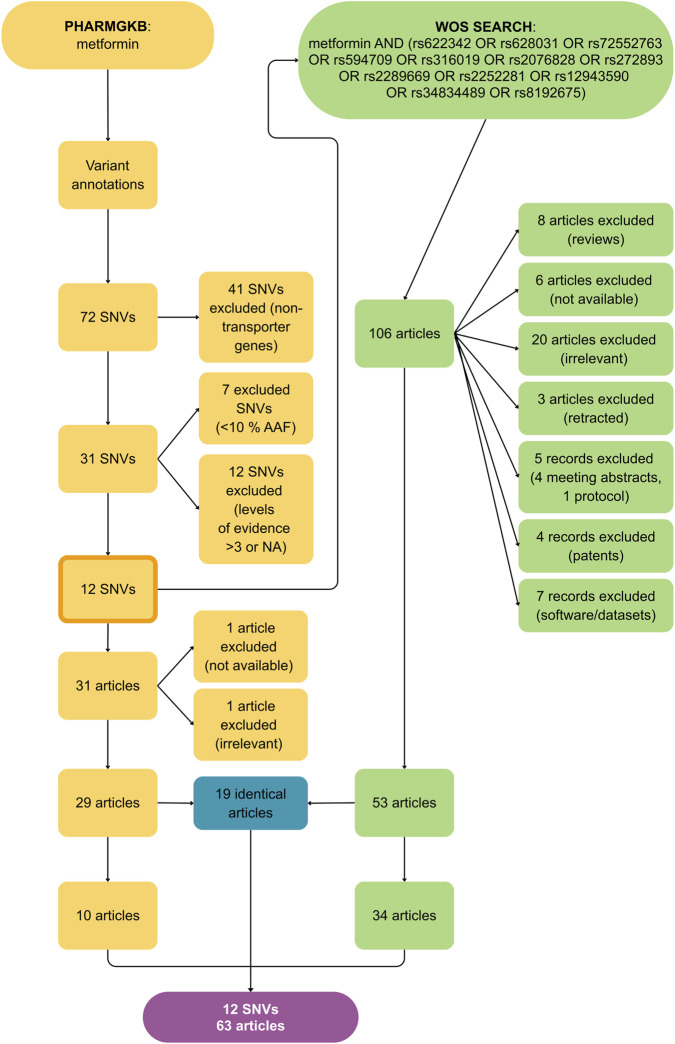
A flowchart showing the procedure of the literature search for single nucleotide variants (SNVs) affecting metformin pharmacogenetics (created in Canva); WOS, Web of Science; SNV, Single nucleotide variant; AAF, Alternate allele frequency.

First, we have identified all SNVs known to be involved in the pharmacokinetics and/or pharmacodynamics of metformin in the Pharmacogenomics Knowledge Base (PharmGKB) database. Only SNVs in transporter genes and with clinical annotation levels of evidence ≤3, according to the PharmGKB database, were included in the final analysis (Level 4 is defined as “unsupported evidence”). For the purposes of this review, only variants common in the European population (in other words, with a sufficient representation to support the sufficient size of study groups in normal-sized studies and, potentially, being clinically meaningful to be determined in this population) were selected, i.e. only SNVs with alternate allele frequency (AAF) > 10%). The AAFs of the analyzed variants were obtained from the National Center for Biotechnology Information (NCBI) SNP database; namely, individual variants were searched using their reference SNP identifiers (rsIDs) *via* the NCBI search interface. After accessing the corresponding variant record, allele frequency data were retrieved from the “Frequency” section of the entry. For this review, the AAF values reported for the European population were used for further analysis. Importantly, we have used the reference allele designation according to the NCBI SNP database, not according to the actual representation of the allele in the population, which might cause some alternate/variant allele frequencies to show actually frequencies over 50% in the European population. This decision was made so that our designation is aligned with this globally acknowledged database. Publications linked to these SNVs were found using the PharmGKB and Web of Science (WOS) databases. The search strategy is illustrated in Figure 1.

### 2.1 PharmGKB database

Data were retrieved from the PharmGKB database (recently, it has been integrated into the ClinPGx resource; our search was, however, performed before that). The PharmGKB database was searched for the word „metformin“ in July 2025, the Variant Annotations tab was used to identify SNVs, yielding 72 single-nucleotide variants. Of these, only SNVs in transporter genes with clinical annotation levels of evidence ≤3, according to the PharmGKB, were selected. Subsequently, only SNVs with an AAF in the European population of >10% (according to the NCBI database) were selected. In all, this search identified 12 SNVs associated with metformin efficacy. For these variants, the specific genes in which the SNVs are located, proteins, alternate allele frequencies, and types of genetic variants were traced and used for the preparation of Table 1. The publications for these 12 SNVs were exported from the PharmGKB database (31 publications in total). Subsequently, one publication was removed because of unavailability (we were unable to retrieve it from available databases), and another one because it was not relevant to this review. In total, 29 publications were used, of which 19 were also found in WOS (see below).

**TABLE 1:** Selected single nucleotide variants (SNVs) associated with metformin therapy found in the PharmGKB database that are common in the European population.

Protein	Gene	rs number	AAF (NCBI)	Type of variant
OCT1	*SLC22A1*	rs622342	C = 0.3643	Intron variant
OCT1	*SLC22A1*	rs628031	A = 0.4036	Missense variant
OCT1	*SLC22A1*	rs72552763	AT = 0.1492	Inframe deletion
OCT1	*SLC22A1*	rs594709	G = 0.4026	Intron variant
OCT2	*SLC22A2*	rs316019	A = 0.1028	Missense variant
OCT3	*SLC22A3*	rs2076828	G = 0.4292	3′UTR variant
OCTN1	*SLC22A4*	rs272893	T = 0.3831	Missense variant
MATE1	*SLC47A1*	rs2289669	A = 0.4165	Intron variant
MATE1	*SLC47A1*	rs2252281	C = 0.3955	5′UTR variant
MATE2-K	*SLC47A2*	rs12943590	A = 0.2802	5′UTR variant
MATE2-K	*SLC47A2*	rs34834489	A = 0.3575	Upstream variant
GLUT2	*SLC2A2*	rs8192675	C = 0.2974	Intron variant

OCT1, Organic cation transporter 1; OCT2, Organic cation transporter 2; OCT3, Organic cation transporter 3; OCTN1, Organic cation/carnitine transporter 1; MATE1, Multidrug and toxin extrusion protein 1; MATE2-K, Multidrug and toxin extrusion protein 2; GLUT2, Glucose transporter type 2; AAF, alternate allele frequency in european population.

Here, it needs to be pointed out that while the rs72552763 is not an SNV in the strict sense of the word (it is a three-nucleotide deletion), the search in PharmGKB returned it among SNVs. After much discussion, we decided to include it in the review despite this discrepancy as it has a relatively high AAF in the European population and is one of the most investigated alterations in the *SLC22A1* gene.

### 2.2 Web of science database

The WOS database was searched in March 2026 using the search string “metformin AND (rs622342 OR rs628031 OR rs72552763 OR rs594709 OR rs316019 OR rs2076828 OR rs272893 OR rs2289669 OR rs2252281 OR rs12943590 OR rs34834489 OR rs8192675)”. This process yielded 106 publications associated with metformin and selected SNVs. Of those, 53 records were removed–namely, we were unable to source six papers (designated as not available in Figure 1), twenty bore no information related to the topic of the review despite being suggested by WOS (designated as “irrelevant”), eight were reviews, four meeting abstracts, one study protocol, four were patents, seven software reports/datasets, and three papers were retracted. Of the remaining 53, 19 were duplicate with PharmGKB. In all, therefore, 63 papers were included in the review.

## 3 Results

**FIGURE 2:**
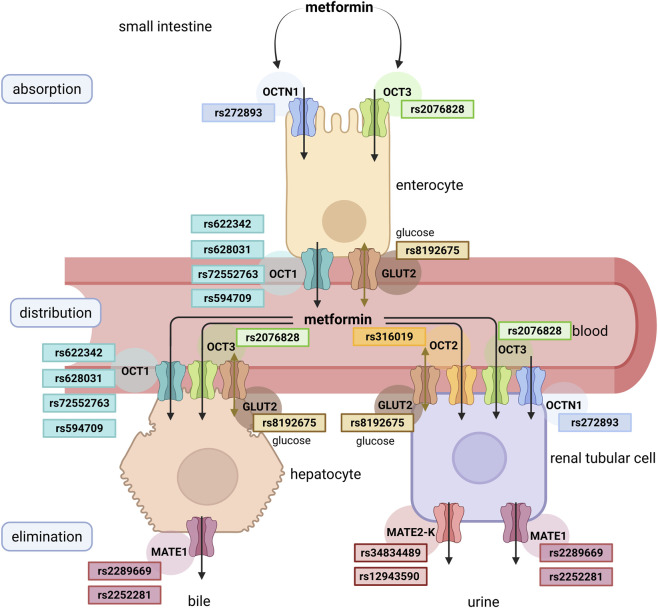
An overview of selected transporter proteins involved in metformin pharmacokinetics in human organism influenced by SNVs discussed in this study. Metformin is taken orally as a hydrochloride and is absorbed in the proximal small intestine (duodenum) (Liang and Giacomini, 2017). The transporter OCTN1 (organic cation/carnitine transporter 1) and OCT3 (organic cation transporter 3) carry it over from the lumen of the intestine to enterocytes (Liang and Giacomini, 2017). Metformin then enters the bloodstream (transported from enterocytes by organic cation transporter 1, OCT1) and is distributed throughout the body (Pérez-Gómez et al., 2023). It does not bind to plasma proteins (Liang and Giacomini, 2017). The OCT1 and OCT3 transporters serve to transport it into hepatocytes and, subsequently, it is excreted into the bile *via* the MATE1 protein (multidrug and toxin extrusion protein 1) (Pérez-Gómez et al., 2023). Besides, metformin is taken up into renal tubular cells by OCT2 (organic cation transporter 2) and OCT3 transporters and is subsequently excreted into the urine by MATE2-K (multidrug and toxin extrusion protein 2) and MATE1 (Wu et al., 2000; Liang and Giacomini, 2017; Pérez-Gómez et al., 2023). The GLUT2 (Glucose transporter type 2) facilitates glucose transport and is highly expressed in the liver, small intestine, and also kidney (Thorens, 2015; Zhou et al., 2016). GLUT2 does not transport metformin directly, nor are there any direct interactions. Created in BioRender. Bořilová Linhartová, P. (2026) https://BioRender.com/4vvb05j SNV, Single nucleotide variant; OCT1, Organic cation transporter 1; OCT2, Organic cation transporter 2; OCT3, Organic cation transporter 3; OCTN1, Organic cation/carnitine transporter 1; MATE1, Multidrug and toxin extrusion protein 1; MATE2-K, Multidrug and toxin extrusion protein 2; GLUT2, Glucose transporter type 2.

Where metformin effectiveness is concerned, the variants in transporter genes are most frequently mentioned in the literature. In particular, these include SNVs in genes for organic cation transporter 1 (OCT1), organic cation transporter 2 (OCT2), organic cation transporter 3 (OCT3), organic cation/carnitine transporter 1 (OCTN1), multidrug and toxin extrusion protein 1 (MATE1), multidrug and toxin extrusion protein 2 (MATE2-K), glucose transporter type 2 (GLUT2), see Figure 2. Supplementary Table S1 shows an overview of metformin-associated SNVs in transporter genes considered for inclusion in the review.

The selection procedure yielded a total of 12 SNVs in metformin transporters eligible for inclusion in the review, specifically variants in the solute carrier family 22 member 1 (*SLC22A1*), solute carrier family 22 member 2 (*SLC22A2*), solute carrier family 22 member 3 (*SLC22A3*), solute carrier family 22 member 4 (*SLC22A4*), solute carrier family 47 member 1 (*SLC47A1*) genes, solute carrier family 47 member 2 (*SLC47A2*) and genes solute carrier family 2 member 2 (*SLC2A2*).

Please note that even though the frequencies of the reference and variant alleles presented for each variant are valid for European population, studies from populations with other ancestries were also included to get a larger pool of knowledge on the effects of individual variants on metformin action.

### 3.1 SNVs in the OCT1 transporter (*SLC22A1* gene)

OCT1 is primarily involved in hepatic uptake of metformin, while its role in intestinal transport remains under discussion (Figure 2). SNVs leading to defective OCT1 may be, due to its reduced hepatic uptake, associated with reduced metformin action at the level of hepatocytes and its excretion through hepatocytes (although OCT3 is known to be able to partially substitute the function of defective OCT1; (Nies et al., 2009). Also, its accumulation in enterocytes can lead to increased risk of intestinal adverse effects of metformin.

#### 3.1.1 rs622342

referenceallele C–frequency=36.43%;variantallele A–frequency=63.57%

The rs622342 variant, located in the intron of the *SLC22A1* gene, does not alter the amino acid sequence of the OCT1 protein. However, it has been associated with a lower transcription rate, reducing its amount in the tissue and, therefore, with lower OCT1 activity (Becker et al., 2010; Moreno-González et al., 2025). Where associations of this SNV with changes in HbA1c levels are concerned, reported results are conflicting; while several studies, including a meta-analysis by Dujic et al., did not demonstrate any significant association (Tkáč et al., 2013; Dujic et al., 2017; Out et al., 2018; Phani et al., 2018), others confirmed the association, often with varying impacts on metformin efficacy depending on allele or genotype. Most of these studies associate the minor allele C with poorer glycemic control (higher HbA1c) or lower reduction of HbA1c levels after metformin therapy in comparison to allele A (Becker et al., however, reported only a combined effect with the rs2289669 variant in MATE1) (Becker et al., 2009b; 2010; Umamaheswaran et al., 2015; Ebid et al., 2019; Reséndiz-Abarca et al., 2019; Naja et al., 2020; Ortega-Ayala et al., 2022; 2024a; Orteg-Ayala et al., 2024b). On the other hand, some studies associated the minor allele C with better glycemic control (lower HbA1c) (Wu et al., 2020; Mohammadi Jouabadi et al., 2024; Moreno-González et al., 2025). This variability appears to be population-dependent and might be associated with linkage disequilibrium with other SNVs.

Where metformin pharmacokinetics is concerned, Christensen et al. reported healthy homozygotes with the CC genotype to have lower metformin renal clearance and secretory clearance compared to the AA genotype (Christensen et al., 2013).

#### 3.1.2 rs628031

referenceallele A–frequency=40.36%,variantallele G–frequency=59.64%

The rs628031 (Met408Val) variant is a missense variant in the *SLC22A1* gene. It is generally associated with normal metformin uptake and has no impact on OCT1 expression in *in vitro* tests (Shu et al., 2003), although some studies suggest that the minor allele may slightly reduce transport activity or affect hepatic uptake *in vivo* (Ningrum et al., 2022; Moreno-González et al., 2025). Numerous studies across various ethnic groups—including Egyptian, Chinese, Slovenian, Iranian, Mexican, and Latvian populations—have found no statistically significant association of HbA1c levels with the rs628031 variant after metformin treatment (Tarasova et al., 2012; Klen et al., 2014; Shokri et al., 2016; Chen et al., 2022; Ahmed et al., 2023; Mohammadi Jouabadi et al., 2024; Ortega-Ayala et al., 2024a).

However, other studies have reported associations, such as increased insulin sensitivity in patients with PCOS with allele A (Chang et al., 2019), elevated HbA1c levels (AA genotype) (Reséndiz-Abarca et al., 2019; Singh et al., 2023), or, contrary, greater reductions in that parameter (with the same, AA, genotype) as well as in fasting plasma glucose (GG genotype) (Zhou et al., 2015) after metformin therapy. Interestingly, patients with both homozygous rs628031 genotypes (GG as well as AA) were shown to be 2.7 times more likely to have inadequate glycemic control (HbA1c ≥ 7%) compared with the heterozygous GA genotype (Moreno-González et al., 2025). On the other hand, Altall et al. associated carriers of both GG and GA with higher levels of HbA1c under metformin (or other antidiabetic drugs) treatment compared to AA carriers (Altall et al., 2019). Yet another study associated the allele G with a greater decrease in HbA1c level after metformin treatment than in allele A carriers (Shikata et al., 2007). The high variability of results in those studies suggests that other, so far unknown, factors play a role in the association of this SNV with metformin effectiveness (which is also supported by the aforementioned normal metformin uptake *in vitro*) (Shu et al., 2003). Positive correlation between calcium intake and HbA1c levels was reported in carriers of the allele A on metformin monotherapy but not in allele G carriers (Zepeda-Carrillo et al., 2022).

Ahmed et al. associated the GA or AA genotypes in this variant with increased risk for gastrointestinal tract (GIT) side effects after metformin therapy compared to the GG genotype (Ahmed et al., 2023). This is consistent with findings by Tarasova et al., who associated allele A with GIT adverse effects after metformin therapy (Tarasova et al., 2012). This could be linked to the accumulation of metformin in enterocytes caused by its reduced excretion due to reduced transport activity of OCT1 with allele A (Ahmed et al., 2023). Ningrum et al. found 1.35-fold higher maximum steady-state plasma concentrations of metformin in the G allele (GA and GG) carriers compared to AA and suggested that the maximum dose should be lower in the G allele carriers (Ningrum et al., 2022). These findings are consistent with the expected easier transport from the enterocytes to the bloodstream. Moreover, they proposed a longer interval between metformin administrations in GG carriers to avoid adverse reactions (Ningrum et al., 2022).

#### 3.1.3 rs72552763

referenceGAT–frequency=85.08%,variantdel–frequency=14.92%

The rs72552763 (Met420del) variant is an inframe deletion, reduced-function variant that leads to weakened OCT1 transporter activity and decreased hepatic uptake of metformin (Sundelin et al., 2017; Pedersen et al., 2018; Ortega-Ayala et al., 2024a). However, the physiological implications are not completely clear. While some studies (Tarasova et al., 2012; Dujic et al., 2017; Ortega-Ayala et al., 2022; Ahmed et al., 2023; Pérez-Gómez et al., 2023; Gómez-Hernández et al., 2025; Moreno-González et al., 2025) did not find any statistically significant association between this variant and changes in HbA1c levels, other studies reported an association of genotypes with HbA1c levels in patients with metformin monotherapy. Namely, the GAT/GAT genotype was associated with lower HbA1c levels compared with other genotypes (del/GAT or del/del) (Ortega-Ayala et al., 2024a; Ortega-Ayala et al., 2024b). All three studies confirming this association are, however, studies published by the same group investigating a specific study population (Mexican-Mestizo patients), which suggests population-specific effects, and their results are in contrast with an Ethiopian study showing an increased responsiveness to metformin in patients with the del/GAT allele than in the GAT/GAT genotype (Degaga et al., 2023). The authors of the Ethiopian study, were not able to confirm these results on the allele level; however, the results show a trend towards the del/GAT allele being associated with better responsiveness.

Ahmed et al. found del/GAT carriers to be more susceptible to metformin adverse effects than GAT/GAT carriers on combined metformin/sulfonylurea therapy (Ahmed et al., 2023). As this SNV is known to negatively affect the function of OCT1, this can, as in the case of the previous SNV, be associated with the accumulation of metformin in enterocytes. Other studies evaluated the influence of this variant in combination with other SNVs. For example, in a study evaluating the combination of rs72552763 (M420del) and rs12208357 (R61C) variants, Dujic et al. associated the number of reduced-function alleles in these two OCT1 SNVs with increased odds of metformin gastrointestinal side effects (Dujic et al., 2016). Using ^11^C-metformin positron emission tomography/computed tomography, Sundelin et al. revealed impaired hepatic uptake in carriers of the variants rs72552763 and rs12208357 (Sundelin et al., 2017). Christensen et al. found a significant decrease in the plasma steady-state metformin concentration with an increasing number of reduced-function haplotypes for the four known reduced-function alleles, i.e., rs72552763 (M420del), rs12208357 (R61C), rs34130495 (G401S), and rs34059508 (G465R) (Christensen et al., 2011).

#### 3.1.4 rs594709

referenceallele G–frequency=40.26%,variantallele A–frequency=59.74%

The rs594709 intron variant is associated with changes in acylcarnitine levels, which may affect fatty acid oxidation and insulin resistance. It is also in linkage disequilibrium with a variant rs113569197, which may possibly influence splicing-altered OCT1 transporter function and reduce metformin efficacy (Kim et al., 2017; Reséndiz-Abarca et al., 2019). The studies by Ortega-Ayala et al. did not find any statistically significant association between the rs594709 variant and changes in HbA1c in Mexican population (Ortega-Ayala et al., 2022; 2024a; Ortega-Ayala et al., 2024b). It is, however, unclear how many patients with individual genotypes were included in the study; the studies comprised populations of 103, 59, and 69 patients, respectively, with only the last one showing the number of participants with individual genotypes (AA = 50, AG = 18 a GG = 1, which is, with respect to the GG homozygote, insufficient for any statistical evaluation). This suggests that the study power in the remaining two studies, in which the genotypes were not detailed, might not have been sufficient to detect any possible differences, either. On the other hand, Reséndiz-Abarca et al. identified, also in a Mexican population with T2D, a significant association of the GG-rs594709 genotype with increased HbA1c levels after 12 months of metformin treatment (Reséndiz-Abarca et al., 2019). No association of rs594709 with adverse drug reaction after metformin treatment in T2D patients was detected (St-Amour et al., 2025).

### 3.2 SNVs in the OCT2 transporter (*SLC22A2* gene)

The OCT2 transporter participates in metformin clearance by facilitating its transport from blood into renal tubular cells. Therefore, variants leading to defective OCT2 could lead to increased blood levels of metformin, which could improve its effectiveness but, on the other hand, might also increase the risk of (rare) systemic adverse effects, such as MALA, vitamin B12 deficiency, coagulation alteration, or hypoglycemia (Shurrab and Arafa, 2020).

#### 3.2.1 rs316019

referenceallele A–frequency=10.28%;variantallele C–frequency=89.72%

The rs316019 variant (Ala270Ser) in the *SLC22A2* gene is a missense mutation and alters the amino acid sequence of the OCT2 protein, which affects its metformin transport function (Abrahams-October et al., 2025). Zolk et al. found in their *in vitro* study on HEK293 cells that the amino-acid change does not affect the expression or membrane localization of OCT2. However, they also associated compound-specific alterations in transport function in cells with allele A compared to the majority allele C (Zolk et al., 2009). Nevertheless, the overall functional consequences remain controversial in the literature. Several studies, including the MetGen meta-analysis with 7,968 T2D patients of European ancestry (Dujic et al., 2017), have not identified any significant association between the rs316019 variant and changes in HbA1c (Tarasova et al., 2012; Stocker et al., 2013; Tkáč et al., 2013; Reséndiz-Abarca et al., 2019; Chen et al., 2022; Ortega-Ayala et al., 2022; 2024a; Pérez-Gómez et al., 2023; Ortega-Ayala et al., 2024b). Phani et al. associated the combination of two variants, rs316019 (CC genotype; in their study, it was presented as GG genotype based on the reverse strand) and rs12943590 in the *SLC47A2* gene (GA genotype) with a decrease in HbA1c levels in incident metformin users (Phani et al., 2018). Another study conducted on transfected HEK293 cells demonstrated higher active metformin uptake in haplotype 2 (rs316019) in comparison with the reference haplotype (Abrahams-October et al., 2025).

In a combined analysis of SNVs rs316019 and rs316009 (another OCT2 SNV), CC/CC homozygotes with T2D were 7.33 times more likely to have adverse reactions to metformin than individuals with reference alleles; these two variants are in linkage disequilibrium (St-Amour et al., 2025). On the other hand, a Korean study evaluating the effects of rs316019 alone found that heterozygotes with the CA genotype had higher maximum peak plasma concentration and higher area under the serum concentration–time curve of metformin in comparison to carriers of the CC genotype (Yoon et al., 2013). This SNV did not affect renal clearance nor secretory clearance when evaluated separately in their study. Another study on the Korean population, however, showed decreased renal clearance in healthy subjects with this variant. This is, however, in contrast with a study on a Danish population (Christensen et al., 2013), where a higher renal clearance and secretory clearance were found for a combination of rs316019 (CA or AA) and rs2252281 (TT) compared with rs316019 (CA or AA) and rs2252281 (TC) present in MATE1. This indicates that renal transport indeed results from an interplay among multiple genes and that isolated evaluation of individual variants may not be sufficient to predict the true phenotype.

### 3.3 SNVs in the OCT3 transporter (*SLC22A3* gene)

The OCT3 transporter participates in metformin absorption from the intestine into enterocytes, and its transport from blood into hepatocytes and renal tubular cells (Figure 2). Thus, defective OCT3 can be expected to both reduce metformin uptake into the organism and its excretion, with effects at the level of hepatocytes likely to follow basically the same trend as effects observed for defective OCT1 (though, possibly, of different magnitude).

#### 3.3.1 rs2076828

referenceallele C–frequency=57.08%,variantallele G–frequency=42.92%

The rs2076828 variant is characterized by a change from the C to G variant in the 3′-UTR region (3′untranslated region) (Chen et al., 2015). The minor G allele of this SNV was associated with reduced expression of the *SLC22A3* gene compared to the reference allele in HCT-116, DU145, and A549 cell lines (Chen et al., 2015). In the same study, lower response to metformin was also observed in healthy volunteers (Chen et al., 2015). This could be explained by the lower absorption of metformin into enterocytes if expression of OCT3 is reduced. However, the studies by Mohammadi Jouabadi et al. and Ortega-Ayala et al. did not demonstrate any significant association between this variant and changes in HbA1c levels (Ortega-Ayala et al., 2022; 2024a; Mohammadi Jouabadi et al., 2024; Imangaliyeva et al., 2025; Ortega-Ayala et al., 2024b). Still, it is important to note that three of those studies did not have a sufficient number of GG homozygotes to meaningfully evaluate the possible effects and the fourth, much larger, study (1,069 participants), did not state the numbers of individual genotypes. No association between rs2076828 and the adverse drug reaction to metformin was found by St. Amour et al. (St-Amour et al., 2025).

### 3.4 SNVs in the OCTN1 transporter (*SLC22A4* gene)

The OCTN1 facilitates the absorption of metformin from the intestine into enterocytes and from blood into renal tubular cells. Impaired function of the OCTN1 transporter is, therefore, likely to both reduce the metformin uptake into the organism and its excretion through the urine.

#### 3.4.1 rs272893

referenceallele T–frequency=38.31%,variantallele C–frequency=61.69%

The rs272893 missense variant leads to an amino acid change from threonine to isoleucine (T306I) (Dujic et al., 2017). Neither Pérez-Gómez et al. (2023) nor a meta-analysis by Dujic et al. found any significant effects of this variant on glycemic response to metformin (HbA1c reduction) in European cohorts, not even after patient stratification (Dujic et al., 2017). In contrast, Vohra et al. found that the C allele in the rs272893 variant was significantly associated with improved response to metformin in Indian patients (reduction in HbA1c). Given these discrepancies, further research on this variant with respect to metformin is needed (Vohra et al., 2023).

### 3.5 SNVs in the MATE1 transporter (*SLC47A1* gene)

The MATE1 transporter is associated with the excretion of metformin through both bile and urine (Figure 2). Reduced function of MATE 1, therefore, could be expected to be associated with improved metformin efficiency (longer half-time of metformin in the organism) but increased risk of adverse effects (Stocker et al., 2013; Chaivanit et al., 2018).

#### 3.5.1 rs2289669

referenceallele G–frequency=58.35%,variantallele A–frequency=41.65%

The rs2289669 variant is an intron variant. An *in vitro* experimental study from 2022 found that this variant does not affect the expression or splicing of *SLC47A1* (Kalamajski et al., 2022). The results of studies investigating this variant on metformin efficacy are, however, contradictory. While multiple studies found no association between rs2289669 and changes in HbA1c or glycemic control in patients on metformin (Tarasova et al., 2012; Klen et al., 2014; Xiao et al., 2016; Dujic et al., 2017; Phani et al., 2018; Raj et al., 2018; Reséndiz-Abarca et al., 2019; Chen et al., 2022; Kalamajski et al., 2022; Gómez-Hernández et al., 2025), others reported associations with therapeutic response (Becker et al., 2009a; Becker et al., 2010; Tkáč et al., 2013; He et al., 2015; Liang et al., 2017; Mousavi et al., 2017; Kim et al., 2022; Ahmed et al., 2023; Pérez-Gómez et al., 2023). Interestingly, Imangaliyeva et al. detected highly significant improvement in metformin efficacy for homozygote AA compared to the AG as well as GG genotype carriers (Imangaliyeva et al., 2025). Besides, Xiao et al. found an (albeit minor) interaction effect of the A allele on the decrease in total cholesterol and low-density lipoprotein cholesterol level compared to the GG genotype, but no influence of this variant on glycemic parameters) (Xiao et al., 2016). Out et al. observed better response to metformin treatment (improved Z scores–change in HbA1c and daily dose of insulin) in carriers of the G allele of rs2289669 compared to allele A (Out et al., 2018). This was consistent with findings by Ningrum et al., who positively correlated the variant allele A with higher fasting blood glucose compared to allele G in patients on metformin (Ningrum et al., 2022). Interestingly, however, Mousavi et al. associated the AG genotype with the greatest reduction in HbA1c after metformin treatment compared to both AA and GG genotypes (Mousavi et al., 2017).

The AA allele was associated with significantly higher odds (p < 0.0001, 95% CI 3.09–8.01) of developing MALA (Chaivanit et al., 2018). On the other hand, no associations of this variant with adverse drug reactions to metformin were observed in Canadian population (St-Amour et al., 2025). Similarly, no influence on pharmacokinetic parameters (such as AUC, Cmax, renal clearance, and others) were observed in healthy individuals in Denmark (Christensen et al., 2015) or in Korea (Moon et al., 2018).

#### 3.5.2 rs2252281

referenceallele T–frequency=60.45%,variantallele C–frequency=39.55%

The rs2252281 variant is a promoter variant (−66T>C) located in the 5′-UTR (5′untranslated region). It may, therefore, potentially affect the transcriptional activity (Ando et al., 2016; St-Amour et al., 2025). However, Ando et al. did not demonstrate any significant difference in MATE1 mRNA levels in peripheral blood cells between individuals carrying the rs2252281 variant and those without it (Ando et al., 2016). Similarly, Mahommadi Jouabadi et al. found in their relatively large study no significant association between the rs2252281 variant and changes in HbA1c levels after metformin treatment (Mohammadi Jouabadi et al., 2024) and similarly, Gómez-Hernández et al. found no association between this polymorphism and metformin treatment efficacy in Mexican patients (Gómez-Hernández et al., 2025). The meta-analysis by Dujic et al. did not reveal any associations of this SNV with metformin effects, either (Dujic et al., 2017). This is consistent with the findings by Christensen et al. who did not find any effect of rs2252281 on the pharmacokinetics of metformin (Christensen et al., 2015). Where lipid metabolism is concerned, Pederson et al. did not associate rs2252281 with any changes in the lipid profile in women with PCOS after metformin therapy, but the number of participants in their study was very low (40 women, only 6 variant allele homozygotes) (Pedersen et al., 2018).

In contrast, the study by Stocker et al. on ethnically diverse population of Californian volunteers associated the allele C with a greater response to metformin in patients with T2DM compared to allele T, which was reflected in a significantly larger relative change in HbA1c levels (patients carrying one or more OCT1 reduced-function variants were removed from the analysis) (Stocker et al., 2013). This is partially corroborated by a pharmacokinetic study reporting lower metformin clearance in carriers of the TC than in those with the TT genotype (note this was observed only in combination with the SNV rs316019, CA or AA genotypes) (Christensen et al., 2013).

### 3.6 SNVs in the MATE2-K transporter (*SLC47A2* gene)

The MATE2-K transporter is associated with metformin excretion through the renal cells. Any decrease in its expression or effectiveness could then be associated with improved metformin action and, potentially, increased risk of systemic or renal adverse effects (Stocker et al., 2013).

#### 3.6.1 rs12943590

referenceallele G–frequency=71.98%,variantallele A–frequency=28.02%

The rs12943590 variant (−130G>A) is a 5′-UTR variant associated with increased promoter activity (Choi et al., 2011; Chung et al., 2013). Some studies have demonstrated an association between this variant and the response to metformin: individuals homozygous for the variant A allele showed a weaker response than GG and GA carriers [e.g., smaller reduction in HbA1c (Choi et al., 2011; Stocker et al., 2013)], while others observed a better response in carriers of the GA genotype (Phani et al., 2018; Raza et al., 2025). Results of these studies are consistent with the hypothesis that the increased promoter activity leads to faster metformin clearance from the organism. Pérez-Gómez only partially agreed with those results, reporting that the AA genotype was associated with lower reduction in blood glucose after 12 months of metformin therapy compared to other genotypes; this was, however, not confirmed for HbA1c levels (Pérez-Gómez et al., 2023). Other studies found no significant association between the rs12943590 variant and HbA1c reduction (Dujic et al., 2017; Raj et al., 2018; Chen et al., 2022; Naem et al., 2024; Gómez-Hernández et al., 2025; Imangaliyeva et al., 2025), other clinical parameters of glucose response (Dujic et al., 2017; Pedersen et al., 2018; Chen et al., 2022; Naem et al., 2024; Gómez-Hernández et al., 2025; St-Amour et al., 2025), or metformin pharmacokinetics (Yoon et al., 2013; Christensen et al., 2015; Moon et al., 2018; Pedersen et al., 2018; Santoro et al., 2018).

#### 3.6.2 rs34834489

referenceallele G–frequency=64.25%,variantallele A–frequency=35.75%

The rs34834489 variant (−396G > A) was *in vitro* associated (in a haplotype combination with rs758427) with a significant increase in reporter gene activity, which was confirmed *in vivo* by enhanced renal clearance of metformin, suggesting increased expression or function (Chung et al., 2013). However, Chung et al. (2013) as well as Gómez-Hernández et al. (2025) found no significant effect of the MATE2-K genotype (including rs34834489) on the glucose-lowering activity of metformin in healthy volunteers (Chung et al., 2013; Gómez-Hernández et al., 2025). St-Amour reported that this SNV was not associated with adverse drug reactions (such as nausea or diarrhea) (St-Amour et al., 2025). No difference in pharmacokinetic parameters between genotypes were observed in Korean population, either (Moon et al., 2018).

### 3.7 SNVs in the GLUT2 transporter (*SLC2A2* gene)

The GLUT2 transporter is not known to transport metformin or interact with it directly. However, it may affect the results of metformin treatment through pharmacodynamic mechanisms–its reduced expression or function leads to lower transport of glucose into the hepatocytes and pancreatic β-cells and, therefore, to higher HbA1c baseline levels. This is, however, successfully corrected by metformin use (but not by sulfonylurea), suggesting a currently unknown relationship between metformin action and this variant (although several hypotheses have been proposed) (Thorens, 2015; Zhou et al., 2016).

#### 3.7.1 rs8192675

referenceallele T–frequency=70.26%,variantallele C–frequency=29.74%

The rs8192675 variant is an intron variant, leading to a reduced expression of the *SLC2A2* gene. The CC genotype is known to show the lowest GLUT2 expression and the TT genotype the highest (Zhou et al., 2016; Wang W. et al., 2024). The MetGen Consortium study demonstrated an association of the C allele with greater HbA1c reduction after metformin treatment (Zhou et al., 2016). In newly diagnosed T2DM patients on metformin monotherapy, allele C was associated with an improved glucose response (reduced fasting glucose) compared to allele T (Rathmann et al., 2019). In contrast, other studies, including a large analysis of 1,069 metformin users from the Rotterdam study (Mohammadi Jouabadi et al., 2024), did not find any statistically significant association between the rs8192675 variant alone and changes in HbA1c levels or therapeutic response to metformin (Pérez-Gómez et al., 2023; Mohammadi Jouabadi et al., 2024). The larger of these two studies, however, detected a strong synergy between rs8192675 and rs7124355 SNVs, which affected both pharmacokinetics (metformin transport) and pharmacodynamics (insulin secretion) (Mohammadi Jouabadi et al., 2024).

## 4 Discussion

Metformin is a drug predominantly prescribed to T2DM patients (Bailey, 2017) (although other indications, including, for example, anticancer treatment (Sato et al., 2025) or treatment of PCOS (Pedersen et al., 2018), are known). However, although it has been proved effective in T2DM treatment, the proportion of non-responders is relatively high, which can be, at least in part, caused by genetic predispositions, such as SNVs in genes encoding transporter proteins affecting metformin pharmacokinetics (and, therefore, the levels of metformin in target tissues) (Rashid et al., 2019).

It has anti-inflammatory (Cameron et al., 2016) and immunomodulatory properties in various immune-related diseases through AMP-activated protein kinase (AMPK)-dependent as well as AMPK-independent mechanisms involving both the innate and adaptive immune systems (Wang Z. et al., 2024). In our study, twelve SNVs in seven genes encoding transporters related to metformin pharmacokinetics (OCT1, OCT2, OCT3, OCTN1, MATE1, MATE2-K, GLUT2) were identified as the most promising for personalized metformin therapy in the European population.

However, further research on the influence of these 12 selected SNVs and their combinations on metformin therapy effectiveness is needed. The analysis of variants associated with drug transport could help in true personalization, i.e., choosing the most suitable treatment for a particular patient–an approach not routinely used so far. Efforts to develop a suitable pharmacogenetic tool for the identification of genetic variants are, however, underway. The work by Xhakaza et al., who evaluated their proposed MassARRAY panel to determine 19 pharmacogenetic biomarkers associated with the treatment of T2DM in an African population, can be mentioned as an example of such an approach (Xhakaza et al., 2020).

However, implementing pharmacogenetic approaches into clinical practice is not easy. Often, there is a lack of prospective randomized clinical trials that would confirm the benefit of pharmacogenomic testing for patients; moreover, neither the optimal clinical algorithm nor the cost-effectiveness of such an approach have been established so far. The issue of reimbursement for pharmacogenetic testing also needs to be resolved. Given the low price of metformin, the current practice rather relies on the iterative approach, i.e., prescribing metformin to a patient and adjusting the dosage based on the outcome during follow-up visits. However, the pharmacogenetic approach could, given the drop in prices of PCR-based pharmacogenetic testing in the wake of the COVID-19 pandemic, gradually become more affordable and, thus, viable. For example, in our laboratories, genotyping of all 12 variants of interest using qPCR would cost approx. 100 Eur per sample, which would likely be reduced in case of routine clinical implementation. Importantly, further research could reduce the number of polymorphisms that need to be reasonably tested. On the other hand, such an analysis could help prevent serious adverse effects in predisposed individuals and improve the likelihood of prescribing suitable dose that would correct the glucose and HbA1c levels at first attempt. Alternatively, genotyping could be performed only in individuals in whom first prescription of metformin did not lead to desired results. Still, the path to the routine clinical use of pharmacogenetic approach leads through large-scale prospective studies, which are currently not available for the entire complex of SNVs proposed to be important in the European population.

The issues of bringing of pharmacogenetic approaches to the clinical practice are being addressed by the Clinical Pharmacogenomics Implementation Consortium (CPIC), which identifies barriers that may arise in implementing a pharmacogenetic approach, and proposes realistic solutions to address these barriers (Pawlyk et al., 2014).

### 4.1 Variability in results of available studies

There are other obstacles, too. In this article, based on a review of pharmacogenetic literature on metformin, we aimed to identify SNVs in metformin transporter genes. However, as shown in Table 2, Supplementary Table S2, the effects of individual variants on the phenotype, i.e., the results of transporter functionality studies, were not consistent and, therefore, it is difficult to draw any firm conclusions. Generally, it seems that rs2289669 and rs622342 are the only variants for which the results appear to be conclusive and have been proven even in relatively small population samples. This can be caused by the combination of relatively strong effect of the variant alleles and their relatively high frequency in population (approximately 42% and 36% in the European population, respectively), which allowed a sufficient number of participants for reasonable statistical power even in small groups of approx. 100 individuals. The study of other variants with much lower AAFs brings challenges from the perspective of costs and feasibility (the necessity to test a large number of individuals to achieve sample sizes with sufficient statistical power), which may be the reason for the lack of positive results in many studies. From this perspective, we dare say that the number of studies reporting lack of differences in metformin action with respect to individual variants should be interpreted with caution as such results can be often caused exactly by the insufficient statistical power for valid comparison (see Supplementary Tables S2, S3 detailing, among other information, numbers of subjects).

**TABLE 2:** Synthesis of the current knowledge on the effect of individual variants in genes encoding transporter proteins on HbA1c levels after metformin treatment for populations worldwide (left part) and European populations (right part).

​	​	Metformin effectiveness								Metformin effectiveness					
​	​	Populations worldwide			European populations			​	​	Populations worldwide			European populations		
SNV	Genotype/Allele	Reduced (↑ HbA1c)	No effect	Increased (↓ HbA1c)	Reduced (↑ HbA1c)	No effect	Increased (↓ HbA1c)	SNV	Genotype/ Allele	Reduced (↑ HbA1c)	No effect	Increased (↓ HbA1c)	Reduced (↑ HbA1c)	No effect	Increased (↓ HbA1c)
rs622342	AA	1	0	3	0	0	1	rs272893	CC	0	0	0	0	0	0
	CC	2	0	0	0	0	0		TT	0	0	0	0	0	0
	AC	0	0	0	0	0	0		CT	0	0	0	0	0	0
	A	0	0	1	0	0	0		C	0	0	1	0	0	0
	C	3	4	2	1	3	1		T	0	2	0	0	2	0
rs628031	GG	0	0	0	0	0	0	rs2289669	AA	0	0	5	0	0	3
	AA	1	0	1	0	0	0		GG	0	0	0	0	0	0
	GA	0	0	1	0	0	0		AG	0	0	0	0	0	0
	G	1	0	1	0	0	0		A	0	10	5	0	4	1
	A	0	8	0	0	3	0		G	0	0	0	0	0	0
rs72552763	del/del	0	0	0	0	0	0	rs2252281	CC	0	0	0	0	0	0
	GAT/GAT	0	0	0	0	0	0		TT	0	0	0	0	0	0
	del/GAT	0	0	1	0	0	0		CT	0	0	0	0	0	0
	del	2	8	0	0	4	0		C	0	3	1	0	2	0
	GAT	0	0	0	0	0	0		T	0	0	0	0	0	0
rs594709	AA	0	0	0	0	0	0	rs12943590	AA	2	0	0	0	0	0
	GG	1	0	0	0	0	0		GG	0	0	0	0	0	0
	AG	0	0	0	0	0	0		AG	0	0	2	0	0	0
	A	0	0	0	0	0	0		A	0	7	0	0	2	0
	G	0	3	0	0	0	0		G	0	0	0	0	0	0
rs316019	CC	0	0	0	0	0	0	rs34834489	AA	0	0	0	0	0	0
	AA	0	0	0	0	0	0		GG	0	0	0	0	0	0
	CA	0	0	0	0	0	0		AG	0	0	0	0	0	0
	C	0	0	0	0	0	0		A	0	1	0	0	0	0
	A	0	9	0	0	4	0		G	0	0	0	0	0	0
rs2076828	GG	0	0	0	0	0	0	rs8192675	CC	0	0	0	0	0	0
	CC	0	0	0	0	0	0		TT	0	0	0	0	0	0
	GC	0	0	0	0	0	0		CT	0	0	0	0	0	0
	G	0	5	0	0	1	0		C	0	2	1	0	2	0
	C	0	0	0	0	0	0		T	0	0	0	0	0	0

Note that each study was entered only once for each variant and the table was constructed as reported in individual papers; hence, for example, improved metformin effectiveness for AA genotype and reduced effectiveness for CC genotype and C allele in the rs622342 variant are all consistent results pointing in the same direction.

Studies also vary in the diversity of ethnic groups studied, including Chinese (Liang et al., 2017; Wu et al., 2020), Korean (Yoon et al., 2013), Indian (Vohra et al., 2023), European (Christensen et al., 2011; Tkáč et al., 2013; Dujic et al., 2017; Rathmann et al., 2019), Egyptian (Ebid et al., 2019; Ahmed et al., 2023), Mexican-Mestizo (Ortega-Ayala et al., 2024a), or other cohorts (e.g. (Stocker et al., 2013; Naja et al., 2020; Ningrum et al., 2022; Naem et al., 2024). It is important to keep in mind that both AAFs and/or linkage disequilibriums can greatly influence the outcomes of individual studies. In populations with higher AAFs, it is easier to achieve a study group that contains a sufficient number of minor allele homozygotes to support reasonable statistical evaluation. This supports study power; in effect, studies in such populations are more likely to yield results revealing associations between the respective variant and metformin action. On the other hand, such benefit can be countered by the presence of linkage disequilibriums between the investigated variant and another one acting in the opposite direction, which can then lead to different outcomes. For example, different effects of the rs622342 on metformin action have been reported in different studies. Although it generally appears that the AA genotype is associated with improved effectiveness (and *vice versa*, CC with poorer effectiveness) of metformin in most studies on Caucasians (and even Mexican Mestizo populations) (Becker et al., 2009b; 2010; Reséndiz-Abarca et al., 2019; Ortega-Ayala et al., 2022; 2024a; Ortega-Ayala et al., 2024b), the only study in Chinese population yielded the opposite result. It is, therefore, possible that in Chinese population, the CC genotype is associated with another one that actually improves its effectiveness (for example, by causing reduced clearance, thus increasing metformin levels in target organs).

Studies are not uniform in the health status of individuals, either–most studies were conducted in diabetic patients, but some papers described studies in women with PCOS (Pedersen et al., 2018; Chang et al., 2019), or in healthy individuals (Chung et al., 2013; Christensen et al., 2015). The daily dose of metformin in the studies was not equivalent, either, ranging from 500 (Zhou et al., 2015) to 2,550 mg (Out et al., 2018) per day. Some studies focused on naive patients, i.e., without previous metformin treatment (Tkáč et al., 2013; Dujic et al., 2016; Phani et al., 2018; Singh et al., 2023; Vohra et al., 2023), while others focused on patients on long-term metformin therapy (Wu et al., 2020; Naem et al., 2024; Moreno-González et al., 2025; St-Amour et al., 2025). Besides, the therapy regimens, i.e., metformin monotherapy (Choi et al., 2011; Stocker et al., 2013; Shokri et al., 2016; Xiao et al., 2016; Phani et al., 2018; Chen et al., 2022), combination of antidiabetic therapy with sulfonylurea (Becker et al., 2009b; Klen et al., 2014; Naja et al., 2020; Ahmed et al., 2023), insulin (Out et al., 2018), or other drugs (Ebid et al., 2019) also differed among studies, with drug-drug interactions (including non-antidiabetic drugs) possibly playing a role in metformin pharmacokinetics (Zepeda-Carrillo et al., 2022).

Other issues causing inconsistencies in results include different rules for determining responsiveness to metformin in individual studies. While most studies investigate changes in HbA1c levels [e.g. (Becker et al., 2009a; Liang et al., 2017; Reséndiz-Abarca et al., 2019; Naja et al., 2020; Pérez-Gómez et al., 2023; Ortega-Ayala et al., 2024b)], it is important to take into account whether they are naïve patients or already long-term users; the responsiveness also depends on the baseline value of the HbA1c parameter. Besides, the observation periods and measured parameters also differ among studies. From this perspective, there is no clear-cut time period for which the parameter should be observed, nor is there a clear threshold of the difference in HbA1c levels denoting the patient as a responder. Among other things, the studies also differ in units (e.g., % vs. mmol/mol for HbA1c). Besides HbA1c, other parameters, such as fasting blood glucose e.g. (Umamaheswaran et al., 2015; Zhou et al., 2015; Altall et al., 2019; Chen et al., 2022), postprandial glucose (Umamaheswaran et al., 2015; Zhou et al., 2015; Chen et al., 2022), glucose AUC (area under the curve) from the oral glucose tolerance test (Chung et al., 2013), fasting insulin (Chen et al., 2022), postprandial insulin (Chang et al., 2019), insulin sensitivity indices (Wu et al., 2020; Chen et al., 2022), lipid profile (total cholesterol, low-density lipoprotein cholesterol, triglycerides, very low-density lipoprotein, high-density lipoprotein cholesterol) (Klen et al., 2014; Umamaheswaran et al., 2015; Xiao et al., 2016; Abrahams-October et al., 2021; Ortega-Ayala et al., 2022; Zepeda-Carrillo et al., 2022), and pharmacokinetic (Chen et al., 2015) parameters are commonly measured.

### 4.2 Other factors potentially influencing SNVs’ effects on metformin action

Gene-gene interactions play an important role, as the combination of different variants in different transporter genes can have synergistic but also antagonistic/counteracting effects, depending on where in the organism the transporters with variant alleles are located (Becker et al., 2010; Christensen et al., 2013; Stocker et al., 2013; Mohammadi Jouabadi et al., 2024). As an example, we can refer to the rs622342 where conflicting results were found in different populations. This may indicate that individual variants could be associated with others that have antagonistic effects, preventing simple recognition of the individual variants.

Some studies deliberately exclude patients with variants that might interact with another variant, while others do not (Stocker et al., 2013; Singh et al., 2023). Reduced expression of one transporter may affect the efficiency of another (Dujic et al., 2017). It is also possible that additional, yet unknown, transporters may be involved in metformin transport (Dujic et al., 2017). Some studies have also reported the influence of variants in non-transporter genes on the pharmacokinetics and pharmacodynamics of metformin (Christensen et al., 2015; St-Amour et al., 2025). For this reason, it would be a good start to first perform precise functional studies of individual variants in cell culture models to demonstrate the isolated effects of individual variants. Although several studies of this kind have already been performed (Choi et al., 2011; Chen et al., 2015; Kalamajski et al., 2022; Abrahams-October et al., 2025), more are needed. This could also support the comprehensive investigation of the simultaneous effect of multiple genetic variants on metformin levels.

The inconsistency of entries for the variants poses another challenge. Variants are commonly referred to by rs numbers, base substitutions in certain positions, or amino acid substitutions, and not every paper lists all types of entries for a single variant, which is then confusing and awkward when searching for the variant in the literature. The rs number identifies a specific SNV location, but does not indicate which allele is associated with a phenotypic effect or how it affects function. Further ambiguity may arise from allele designation, as some studies report the change on the forward (positive) strand, while others use the reverse (negative) strand. The issue of metformin pharmacogenetics still has many challenges to overcome, including more high-quality studies, uniform or similar study designs, specification of basic concepts from a clinical point of view (e.g., a definition of responders and nonresponders), and the establishment of generally applicable guidelines for metformin pharmacogenetics. Large GWAS-type studies or meta-analyses that could clarify the clinical relevance of specific variants in patients are also needed.

Of course, when thinking about the implementation of pharmacogenetic approaches in personalization of metformin treatment, it is important to consider, besides genetic factors, also other factors and environmental modifiers such as BMI (Shikata et al., 2007), age (Tarasova et al., 2012), diet (St-Amour et al., 2025), health status (Yoon et al., 2013), lifestyle (St-Amour et al., 2025), alcohol consumption (Tarasova et al., 2012), smoking (Pérez-Gómez et al., 2023), or years since T2D diagnosis (Becker et al., 2009a; Tkáč et al., 2013; Mohammadi Jouabadi et al., 2024). These factors should, of course, also need to be considered in the studies to improve their clinical relevance.

### 4.3 Suggestions for future studies

The great complexity of the pharmacogenetic analyses poses a great challenge for scientists and, as discussed above, for the comparability of studies. For these reasons, we would like to propose the following framework:

- It is important to know the frequency of the variant allele in the particular study population before the study design and target number of patients are decided upon. The frequencies of individual alleles may differ across populations and a low number of patients with the minor allele may lead to an insufficient study power (and, thus, false negative results). This should be accounted for when planning the study to ensure sufficient numbers of participants in all groups;
- The participant group should be as homogeneous as possible from the perspectives of genetic background, health status, previous metformin use (patients previously metformin-naïve can show different responses from long-term metformin users), metformin dose (higher doses may influence the capacity for metformin excretion by exhausting the transporter proteins, which may act as a bottleneck, and mask the true effects of the variant). All these characteristics need to be reported;
- As a minimum, HbA1c levels should be reported (at the baseline and, ideally, at multiple time points, e.g., 3 months and 6 months); reporting additional parameters, including pharmacokinetic parameters or results of additional characteristics of glucose metabolism, is also beneficial;
- To use the rs number of the variant in the publications to ensure the study is not missed in search;
- To report all relevant study group characteristics (age, BMI, health status, numbers of individual genotypes found in the study, ideally also renal function);
- It is necessary to keep in mind possible drug interactions; ideally, only patients on metformin monotherapy should be included in the study;
- To report possible adverse effects of metformin use;
- Ideally, a complete set of SNVs discussed in this paper should be determined as a minimum in future studies as it is obvious that their mutual interplay can affect the responsiveness; therefore, evaluation of only individual SNVs without the knowledge of the complexity of such mutual interactions, it is difficult to accurately predict the effectiveness of metformin therapy in individual patients.
- Many other issues are worth considering when designing such studies, including baseline HbA1c adjustment, treatment duration, adherence assessment, formulation, reporting genotype counts, Hardy-Weinberg equilibrium, ancestry-informative data, and gene-gene/gene-environment interactions.

### 4.4 Limitations

The fact that we did not include all genes with variants that may affect the pharmacogenetics of metformin can be considered a limitation; however, this would excessively inflate the paper; moreover, the selection criteria we applied ensure that the most relevant variants are included. Still, some papers might have been missed if they did not include the rs numbers but used only alternative designations of the variants.

## 5 Conclusion

The single-nucleotide variants with assumed functional consequences in metformin-associated transporter genes play an important role in influencing metformin pharmacokinetics. Thus, they may affect both metformin treatment effectiveness and adverse effects. As metformin is a widely used drug, investigation of the individual responses from a pharmacogenetic perspective might in the future contribute to the personalization of metformin treatment, increasing the efficacy of treatment while minimizing adverse effects. However, there is a need to develop tools to overcome some of the challenges encountered in metformin pharmacogenetic research. Also, prospective validation, clinical algorithms, cost-effectiveness studies, and guideline-level evidence need to be developed before such recommendations can be introduced into clinical practice. The high variability among studies from the perspectives of study design, population, reported characteristics, and other parameters make it difficult to draw firm conclusions. For this reason, we also propose a framework for the design and reporting of pharmacogenetic studies on metformin-associated transporter genes.

## Supplementary material

Supplementary Table S1 The selection of SNVs in metformin-associated transporter genes considered for inclusion in the review based on the PharmGKB database and selection criteria (alternate allele frequency, AAF, in the European population > 10%, Level of Evidence according to PharmGKB ≥ 3).

Supplementary Table S2 Overview of variants investigated in the individual studies included in the review.

Supplementary Table S3 Overview of effects of individual variants on metformin action on HbA1c levels reported in the studies included in the review. Cohort size: small, <100 individuals; medium, 100-500 individuals; large, >500 individuals; T2DM – Type 2 diabetes mellitus.
